# Production of Bioactive Substances to Alleviates Hangover and Ethanol-Induced Liver Damage through Fermentation of *Oenanthe javanica* Using *Lactiplantibacillus plantarum*

**DOI:** 10.3390/molecules27041175

**Published:** 2022-02-09

**Authors:** Da Hye Gam, Jae Hyun Park, So Hee Kim, Min Ho Kang, Se Bin Kim, Jin Woo Kim

**Affiliations:** 1 Department of Food Science, Sun Moon University, Natural Science 118, 70 Sunmoon-ro 221, Tangjeong-myeon, Asan-si 336-708, Korea; ank7895@naver.com (D.H.G.); gengihoo@naver.com (J.H.P.); ssohiii@naver.com (S.H.K.); alsgh5328@naver.com (M.H.K.); 2 Department of Pharmaceutical Engineering & Biotechnology, Sun Moon University, 70 Sunmoon-ro 221, Tangjeong-myeon, Asan-si 336-708, Korea; jun8687@daum.net; 3 FlexPro Biotechnology, Natural Science 128, 70 Sunmoon-ro 221, Tangjeong-myeon, Asan-si 336-708, Korea

**Keywords:** *Oenanthe javanica*, bioconversion, *p*-coumaric acid, hangover, hepatocirrhosis

## Abstract

The purpose of this study is to evaluate the effect of the bioconversion products of *Oenanthe javanica* extract fermented by *Lactiplantibacillus plantarum* (OEFL) on relieving hangovers and improving liver function. In addition, the bioactive substance of the OEFL, which alleviates hangover and ethanol-induced liver damage, was identified and its bioactive property was verified through in vivo experiments. In major substances analysis using high-performance liquid chromatography, OEFL produced 9.5-fold higher *p*-coumaric acid than the *O. Javanica* extract (OE). In addition, considering that quinic acid, which is not present in the OE, was produced in the OEFL it was confirmed that chlorogenic acid was decomposed into quinic acid by bioconversion. In the in vivo experiment using Sprague-Dawley rats, the OEFL and *p*-coumaric acid diets reduced blood ethanol, acetaldehyde, GPT, and ALP concentrations, increasing blood albumin concentrations compared to ethanol-administered groups, demonstrating that OEFL and *p*-coumaric acid, the main substance in the OEFL, improved ethanol-induced liver damage. Furthermore, the OEFL and its main bioactive substance, *p*-coumaric acid, alleviated liver fibrosis by downregulating *TGF-β, SMAD-2, SMAD-4, α-SMA*, and upregulating *MMP-1*. Therefore, OEFL is expected to be used as a functional food or pharmaceutical material as it has been confirmed to effectively relieve hangovers, prevent liver damage, and delay liver fibrosis in ethanol-induced liver damages.

## 1. Introduction

While moderate drinking can provide positive effects, such as stress relief and cardiovascular disease reduction, excessive drinking leads to physical afflictions and psychological problems, such as cancer, liver disease, hangover, depression, dementia, etc. [1]. The hangover is an unpleasant physical and psychological phenomenon that occurs after excessive drinking, presenting symptoms such as headache, diarrhea, fatigue, anxiety, and excitement [2]. Various studies have been ongoing for the development of the hangover remedy that assists ethanol detoxification and alleviation of related symptoms since hangover causes temporary short-term memory, lack of concentration, disorientation, and cognitive dysfunction [3]. However, since most traditional hangover remedies focus only on promoting ethanol oxidation, the recovery of ethanol-induced liver damage is limited. Hence, it is necessary to develop a new functional substance that is effective for relieving hangover as well as improving liver damage [4].

When ethanol is consumed, it is absorbed in the stomach and the small intestine, and then metabolized by an enzyme, alcohol dehydrogenase (ADH) and aldehyde dehydrogenase (ALDH), in the liver. ADH breaks down alcohol into acetaldehyde, and then another enzyme, ALDH rapidly metabolized acetaldehyde into acetate. The acetate is further metabolized, and eventually leaves the body as carbon dioxide and water [5]. Ethanol oxidation occurs in two stages; in the first stage, ethanol dehydrogenase (ADH) oxidizes 80–90% of the absorbed ethanol in the liver to a toxic substance called acetaldehyde (CH_3_CHO), a known carcinogen [6]. The produced acetaldehyde is then oxidized to acetate by aldehyde dehydrogenase (ALDH) and acetate is converted to acetyl coenzyme A, which goes through the tricarboxylic acid cycle to either produce adenosine triphosphate or is utilized in the synthesis of cholesterol and fatty acid [7]. On the other hand, 10–20% of the absorbed ethanol is metabolized by the microsomal ethanol oxidizing system (MEOS) and catalase (CAT). This metabolic pathway reportedly generates reactive oxygen species (ROS) that cause liver damage [8]. Since the oxidative stress engendered by the excessive ROS accelerates liver damage, reducing ethanol oxidation by the MEOS via the activation of ADH helps to protect the liver [9]. The liver diseases caused by ethanol-induced liver damage are fatty liver, hepatitis, and hepatocirrhosis [10]. Among them, hepatocirrhosis is an irreversible disease with a 5-year survival rate of less than 50% and has a high incidence of complications, such as jaundice, ascites, and esophageal variceal hemorrhage; hence, early prevention of ethanol-induced liver damage is imperative [11]. Hepatocirrhosis treatment involves remedy such as steroid and pilin as liver function enhancers; however, their practical effects are ineffective and they have several side effects, including urtication, diarrhea, and enteritis and urgently increasing the need for an alternative remedy [12].

While the effect of natural products, such as raisin tree, bamboo, bean sprouts, and pine needles for relieving hangover or improving ethanol-induced liver damage has been researched to replace the existing synthetic remedy, bioactive substances extracted from natural products may have a different effect because the digestive absorption rate varies by individuals [13]. Therefore, the technology to increase digestive absorption rates of bioactive substances by converting them into smaller molecules or changing the polarity of molecules should be secured [14]. Bioconversion is a technique that utilizes biocatalytic reactions of microorganisms or enzymes to produce low-molecular substances that can be easily absorbed and utilized for various metabolic activities, benefitting human health [15]. The bioconversion by the fermentation of microorganisms is superior to other methods because it has a high degree of substrate specificity, comparatively lower energy consumption, milder reaction conditions, and lower toxic by-product generation [16]. In particular, regarding the bioconversion by the fermentation of microorganisms, the use of probiotics is recognized as a safe process that high survival in intestinal conditions, and it is broadly applied in fields of food, pharmaceutical, and cosmetics [17].

This study sought to develop a functional substance from fermented *Oenanthe javanica* that could effectively relieve hangover and improve ethanol-induced liver damage, which is known to be an antidote and has been used as a medicinal plant to treat a number of liver diseases in Asia [18]. Bioconversion by *O. javanica* fermentation was performed, and the bioconverted *O. javanica* extract (OEFL) with improved bioactive properties was achieved. In addition, through an in vivo experiment using Sprague Dawley (SD) rats, the effects of OEFL relieving hangovers and improving liver function were identified. Then, the expressions of *ADH, ALDH*, and *TGF-β/SMAD*-mediated signaling pathways were evaluated to verify the ethanol oxidation and ethanol-induced hepatocirrhosis inhibition effects of the OEFL, and its industrial applicability was confirmed.

## 2. Results and Discussion

### 2.1. Identification of Bioactive Substances

**Figure 1:**
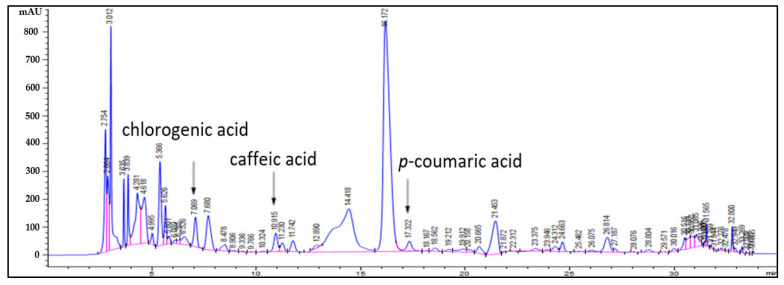
HPLC chromatogram obtained from OE produced by ultrasound-assisted extraction.

**Figure 2:**
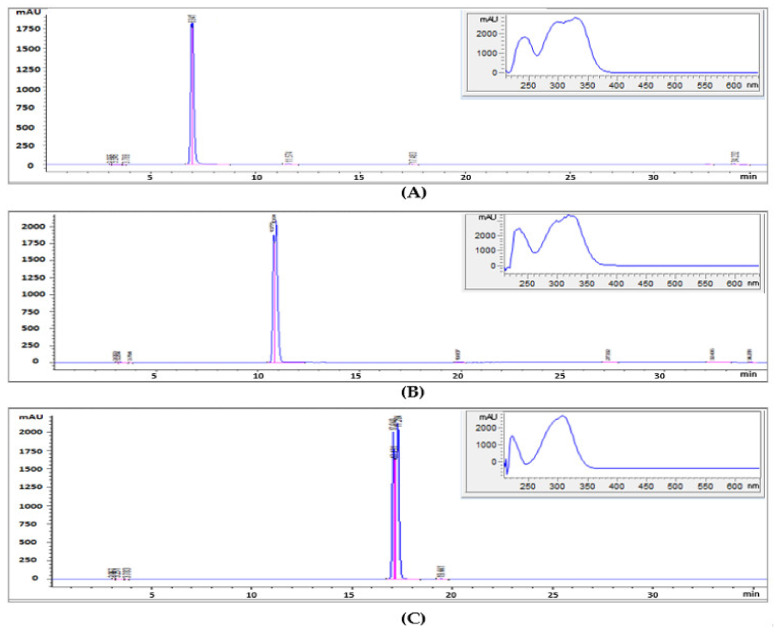
HPLC chromatograms and UV spectra of chlorogenic acid (**A**), caffeic acid (**B**), and *p*-coumaric acid (**C**).

In the analysis of the main components of *O. javanica* extract (OE) using HPLC, the retention times (RT) of the main peaks were 12.4, 16.9, and 24.6 min, consistent with the RT of chlorogenic acid, *p*-coumaric acid, and caffeine among the standard substances (Figure 1 and Figure 2). In addition, as the profiles of the diode-array detection (DAD) spectra were identical with standard substances, the main bioactive substances as polyphenols of OE were reconfirmed to be chlorogenic acid, caffeic acid, and *p*-coumaric acid. The calculated concentrations based on the peak areas were chlorogenic acid, 3.84 mg/g; caffeic acid, 1.07 mg/g; and *p*-coumaric acid, 0.64 mg/g; chlorogenic acid proved to be a main bioactive substance of OE. Chlorogenic acid, an ester of caffeic acid with quinic acid, is one of the most abundant polyphenols in human diets [19]. It has been reported that bioactive property was directly proportional to the concentration of the main substances [20]. Thus, our results suggested that chlorogenic acid was the key substance that affect the bioactive property in OE.

**Figure 3:**
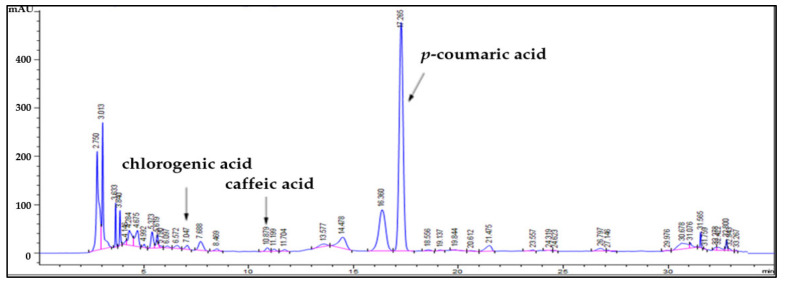
HPLC chromatogram obtained from OEFL.

The main peak of the bioconverted *O. javanica* extract by *Lactiplantibacillus plantarum* (OEFL) was confirmed at 17.2 min (Figure 3) and maximum absorption wavelength was detected at 320 nm in the DAD spectrum, which matched the RT and spectrum of *p*-coumaric acid, one of the standard substances. The concentration of *p*-coumaric acid in OEFL was found to be 6.01 mg/g, which was 9.5-fold higher than OE detected in the previous study (0.64 mg/g). Thus, the bioactive substances of OE were bioconverted through fermentation using *L. plantarum*. In addition, the concentration of *p*-coumaric acid from OEFL was also found to be 2.9-fold higher than the *p*-coumaric acid extracted from *Populus albaglandulosa* in a study by Choi et al. [21]. It is reported that *p*-coumaric acid, identified as a main bioactive substance of the OEFL, reduces oxidative stress-induced DNA damage in cells and has effects on antioxidation, antiinflammation, anticancer, and drop in the blood sugar concentration [22]. Therefore, the enhancement of bioactive properties due to an increase in *p*-coumaric acid in OEFL is expected. On the other hand, the peaks at RT 7.01 and 10.8 min in OEFL were identified as chlorogenic acid and caffeic acid, decreased by 9.8-fold and 8.9-fold compared with OE, respectively. This suggests that during the bioconversion by *L. plantarum*, chlorogenic acid was disintegrated simultaneously into caffeic acid and quinic acid while caffeic acid was subsequently bioconverted into a small molecule such as ferulic acid or other substances [23]. In addition, considering that a peak that was not present in OE was detected at RT 5.7 min, it is hypothesized that the unknown bioactive substance was converted through bioconversion. Therefore, the OEFL by *L. plantarum* produces bioactive substances with increasing concentrations of low molecular polyphenols and improving bioavailability. It is considered that peaks that have not been identified by HPLC need to be identified again through additional analysis using LC-MS-MS in future experiments.

### 2.2. Blood Ethanol Concentration Analysis

**Figure 4:**
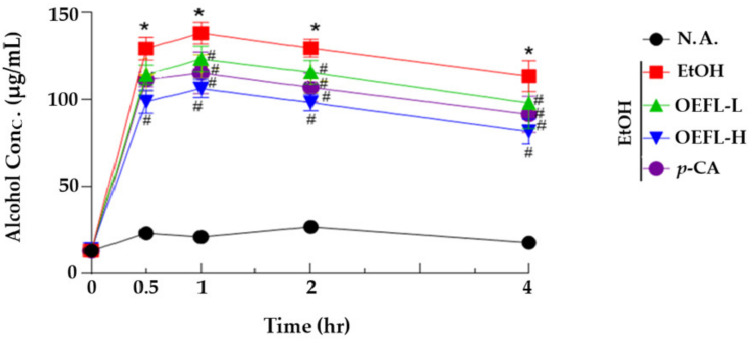
Effects of OEFL and its bioactive substance on blood ethanol concentration in ethanol-administrated SD rats. Rats were orally administrated OEFL or *p*-CA before ethanol administration. N.A.: non-administered group; EtOH: ethanol-administrated group; OEFL-L: OEFL 200 mg/kg-administrated group; OEFL-H: OEFL 600 mg/kg-administrated group; *p*-CA: *p*-coumaric acid 3.6 mg/kg-administrated group; * *p* < 0.05 vs. N.A.; ^#^ *p* < 0.05 vs. EtOH.

To determine the effect of OEFL on ethanol oxidation and ethanol-related toxicity, the blood ethanol concentration was analyzed. The OEFL doses for the experimental groups were 200 and 600 mg/kg, and the *p*-coumaric acid dose was 3.6 mg/kg; thereby the concentration of *p*-coumaric acid was set to the same amount as that in 600 mg/kg of the OEFL (Figure 4). After 0.5, 1, 2, and 4 h of ethanol administration, the blood ethanol concentration decreased in the experimental groups compared with the ethanol-administered group (*p* < 0.05). In the experimental group administered with 200 mg/kg OEFL, the blood ethanol concentration at 4 h after ethanol consumption (98.1 μg/mL) decreased by 1.2-fold compared with the ethanol-administered group (113.4 μg/mL; *p* < 0.05). In the experimental group administered with 600 mg/kg OEFL, the blood ethanol concentration at 2 and 4 h after ethanol administration decreased by 1.2 and 1.3-fold, respectively, compared with the ethanol-administered group (*p* < 0.05). The experimental group administered with 3.6 mg/kg *p*-coumaric acid showed a smaller decrease in blood ethanol concentration compared with the 600 mg/kg OEFL-administered group. It is postulated that various polyphenols, including *p*-coumaric acid, worked in a complex manner in the OEFL-administered experimental groups to promote the activity of ADH and reduce the blood ethanol concentration.

### 2.3. Blood Acetaldehyde Concentration

**Figure 5:**
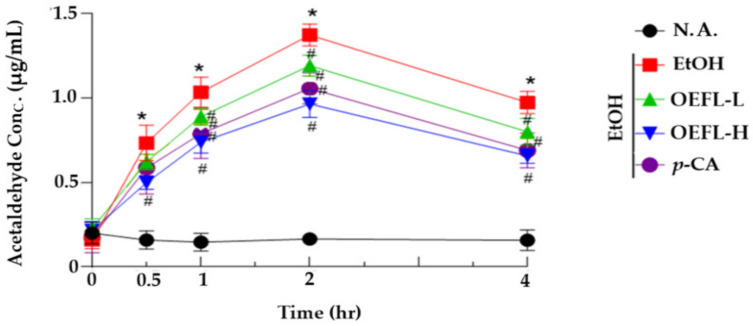
Effects of OEFL and its bioactive substance on blood ethanol concentration in acetaldehyde-administrated SD rats. Rats were orally administrated OEFL or *p*-CA before ethanol administration. N.A.: non-administered group; EtOH: ethanol-administrated group; OEFL-L: OEFL 200 mg/kg-administrated group; OEFL-H: OEFL 600 mg/kg-administrated group; *p*-CA: *p*-coumaric acid 3.6 mg/kg-administrated group. * *p* < 0.05 vs. N.A.; ^#^ *p* < 0.05 vs. EtOH.

Acetaldehyde, an oxidized form of ethanol, is classified as a carcinogen in animal experiments, causing hangovers as well as high cytotoxicity, mutations, and carcinogenicity, so it is important to lower blood concentration to relieve hangovers. Blood acetaldehyde concentration was analyzed to evaluate the efficacy of the OEFL and its bioactive substance for the detoxification of acetaldehyde. In the ethanol-administered group, at 2 h after ethanol administration, the blood acetaldehyde concentration increased to the highest concentration compared with the normal group that administered distilled water (Figure 5). The blood acetaldehyde concentrations after 1, 2, and 4 h of ethanol administration were reduced significantly in the experimental groups (OEFL 200 mg/kg; OEFL 600 mg/kg; *p*-coumaric acid 3.6 mg/kg; *p* < 0.05). In particular, the blood acetaldehyde concentrations at 4 h after ethanol administration in the 600 mg/kg OEFL and 3.6 mg/kg *p*-coumaric acid experimental groups were 0.65 μg/mL and 0.69 μg/mL, respectively, and reduced by 1.5 and 1.4-fold compared with the ethanol-administered group (0.97 μg/mL), respectively. This is a result confirming that OEFL and *p*-coumaric acid increase ALDH activity, effectively detoxicating acetaldehyde to lower blood concentration, thereby alleviating hangovers induced by acetaldehyde.

Acetaldehyde is a toxic substance that leads to hangover and ethanolic liver damage by migrating and accumulating in other organs, including the brain, when the blood acetaldehyde concentration becomes excessive [24]. Yang, et al. reported that black garlic extract was effective in relieving hangover by reducing the concentration of blood acetaldehyde following ethanol administration in acute ethanol SD rat models [25]. In this study, OEFL and its main bioactive substance *p*-coumaric acid are effective in relieving hangovers by enhancing ALDH activity and reducing the concentration of acetaldehyde in the blood, which is the cause of hangover.

### 2.4. Blood GPT, GOT, and ALP Concentrations

The degree of ethanol-induced liver damage was evaluated by determining glutamic pyruvic transaminase (GPT), glutamic oxaloacetic transaminase (GOT), and alkaline phosphatase (ALP) concentrations. GPT and GOT, used as indicators of liver damage, are primarily present in hepatocytes and released into the blood when hepatocytes are damaged, increasing the blood GPT and GOT concentration [26]. In most acute liver diseases, the GPT concentration is higher than or similar to the GOT. GPT is considered a more specific indicator of liver damage than GOT, as GPT is only present in the mitochondria of the liver, so GPT levels are recognized as more important for liver-specific damage [27]. ALP is a protein made by hepatocytes and when hepatocytes are damaged, ALP leaks into the bloodstream, and blood ALP levels increase, which is used to measure liver damage together with GPT and GOT [28].

**Figure 6:**
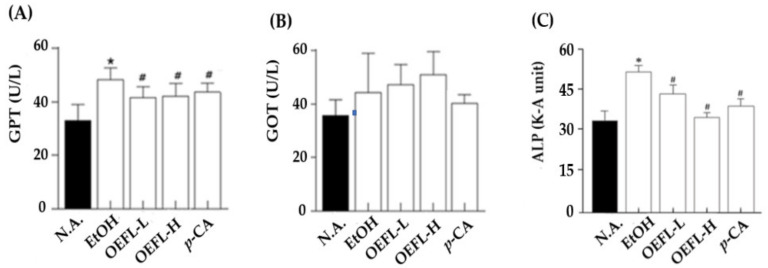
Effects of OEFL and its bioactive substance on (**A**) GPT, (**B**) GOT, and (**C**) ALP concentrations in ethanol-administered SD rats. * *p* < 0.05 vs. N.A.; ^#^ *p* < 0.05 vs. EtOH.

In this study, GPT showed a statistically significant increase in the ethanol-administered group compared with the normal group that collected blood before ethanol administration (Figure 6A). Although the GOT concentrations increased to some degree, it was not statistically significant (Figure 6B; *p* > 0.05). In the 200, 600 mg/kg OEFL, and 3.6 mg/kg *p*-coumaric acid administered experimental groups, the blood GPT concentrations decreased compared with the ethanol-administered group (*p* < 0.05), confirming that OEFL and *p*-coumaric acid could effectively be used in improving ethanolic liver damage. Blood GTP concentrations were similarly measured at 41.7 and 41.4 U/L in the 600 mg/kg OEFL and 3.6 mg/kg *p*-coumaric acid administered experimental groups (*p* > 0.05). It can be predicted from the above-mentioned data that the blood GTP concentrations were reduced due to the cytoprotective effect of *p*-coumaric acid, which is a main bioactive substance contained in OEFL.

Following the administration of ethanol and the experimental substances including OEFL and *p*-coumaric acid, the blood ALP concentration increased by 1.6-fold in the ethanol-administered group (55.4 K-A unit) compared with that of the normal group (33.8 K-A unit; Figure 6C). Ethanol-induced liver damage causes the release of ALP into the blood, increasing blood ALP concentration [29]. Kang et al. reported a 1.2-fold increase in the blood ALP concentrations of ethanol-administered SD rats for 5 weeks compared with the normal group, further confirming that ALP is closely related to ethanolic liver damage [30]. On the other hand, in the 200 and 600 mg/kg OEFL-administered experimental groups, the blood ALP concentrations were different (43.6 and 35.0 K-A units, respectively), indicating that OEFL treatment concentration significantly affected the blood ALP concentration (*p* < 0.05). In particular, the blood ALP concentration of the experimental group administered with 600 mg/kg OEFL be reduced to a level similar to that in the normal group. The experimental group administered with 3.6 mg/kg *p*-coumaric acid also decreased the blood ALP concentration, which had increased due to ethanol administration, to 39.1 K-A unit, confirming that OEFL and *p*-coumaric acid are effective in improving ethanolic liver damage caused by excessive ethanol consumption.

### 2.5. Blood Albumin Concentration

**Figure 7:**
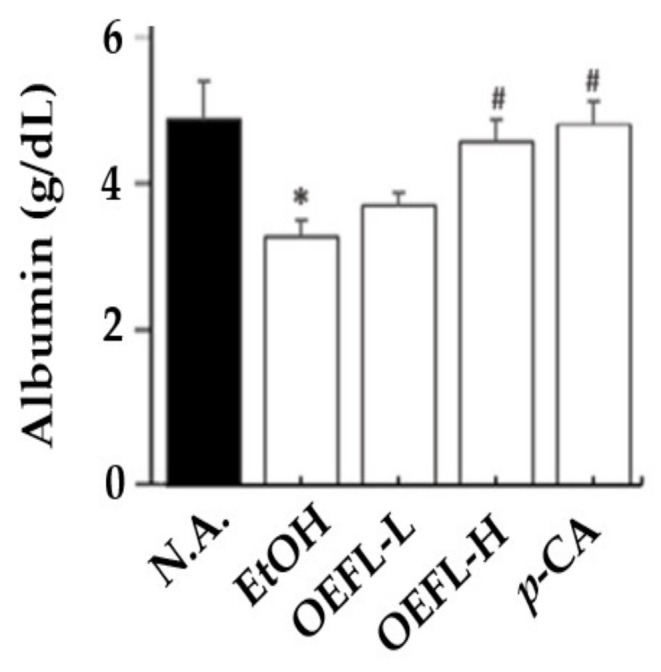
Effects of OEFL and its bioactive substance on albumin concentration ethanol-administered SD rats. * *p* < 0.05 vs. N.A.; ^#^ *p* < 0.05 vs. EtOH.

Blood albumin concentration was analyzed to confirm the degree of liver damage in the ethanol-administered and test groups. It was found that the ethanol-administered group (3.38 g/dL) showed a reduction in blood albumin concentration by 1.4-fold compared with the normal group (4.99 g/dL), indicating that ethanol administration caused ethanolic liver damage (Figure 7). Albumin accounts for 60% of the total blood protein and is specifically synthesized in the liver. Liver malfunction by ethanol-induced hepatocirrhosis will impair the liver’s synthetic role and could have led to decreased levels of albumin. [31]. Therefore, the decrease in blood albumin concentration has a diagnostic indicator in the determination of the chronicity and prognosis of liver diseases; the normal concentration is 3.5–4.5 g/dL [32]. While there was no significant difference in blood albumin concentrations between the ethanol-administered and 200 mg/kg OEFL-administered groups (*p* > 0.05), the 600 mg/kg OEFL and 3.6 mg/kg *p*-coumaric acid administered group showed a higher blood albumin concentration than the ethanol-administered group (*p* < 0.05). In the test groups administered with 600 mg/kg OEFL and 3.6 mg/kg *p*-coumaric acid, the blood albumin concentrations increased by 1.4 and 1.5-fold, respectively, compared with the ethanol-administered group. It was confirmed that the blood albumin concentration was increased to its normal range in the 600 mg/kg OEFL-administered and 3.6 mg/kg *p*-coumaric acid administered groups and ethanolic liver damage was improved. Hence, OEFL and *p*-coumaric acid had a positive effect on ethanolic liver damage by stimulating ethanol metabolism, detoxification, and enhancing albumin synthesis.

### 2.6. Comparison of Gene Expression Related to Ethanol Oxidation in Liver Tissue

**Figure 8:**
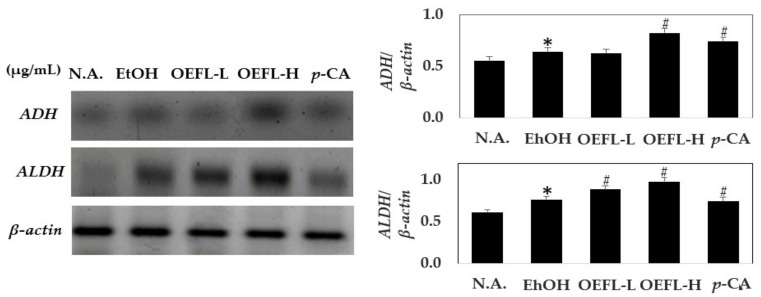
Effects of OEFL and its bioactive substance on expressions of *ADH* and *ALDH* in ethanol-administered SD rats. * *p* < 0.05 vs. N.A.; ^#^ *p* < 0.05 vs. EtOH.

To investigate the mechanism of hangover relief of OEFL, the expressions levels of *ADH* and *ALDH* involved in ethanol oxidation in liver tissues for each experimental group were measured (Figure 8). The expressions of *ADH* and *ALDH* were observed to increase by 1.1-fold in the ethanol-administered group compared with the normal group. In the process of ethanol oxidation, ethanol oxidation is caused by increased *ADH* and *ALDH* expressions. In the 600 mg/kg OEFL-administered group, the expressions of *ADH* and *ALDH* are increased by 1.33 and 1.5-fold, respectively, compared with the ethanol-administered group. OEFL is anticipated to increase ethanol oxidation through oxidation by *ADH* and *ALDH*. In the test group administered with 3.6 mg/kg *p*-coumaric acid, which is the main substance of OEFL, the expressions of *ADH* and *ALDH* increased compared with the ethanol-administered group, confirming ethanol and acetaldehyde oxidation [33]. However, the expressions of *ADH* and *ALDH* were approximately 1.2-fold lower in the 3.6 mg/kg *p*-coumaric acid-administered group compared with the 600 mg/kg OEFL-administered group, which is most likely because bioactive substances present in OEFL, especially *p*-coumaric acid, contributing to the enhancement of *ADH* and *ALDH* activity. Hence, it was confirmed that OEFL had a positive effect on ethanol oxidation and the detoxification of acetaldehyde in liver tissues.

### 2.7. Comparison of Expression of Genes Related to Fibrosis

Ethanol and acetaldehyde can destroy the microtubule structure in hepatocytes which then leads to immune cell infiltration, hepatocyte necrosis and inflammation of the liver, abnormal DNA repair, and mitochondrial damage [34]. All these ethanolic liver damage mechanisms stimulate excessive extracellular matrix (ECM) protein deposition to replace dead hepatocytes which, in turn, causes hepatocirrhosis [35].

**Figure 9:**
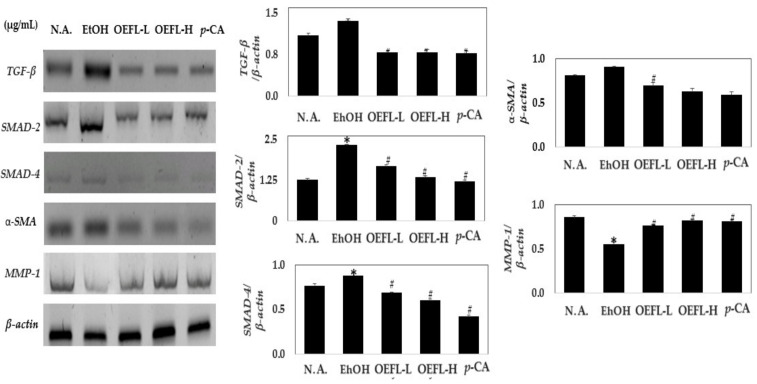
Effects of OEFL and its bioactive substance on expressions of *TGF-β*, *SMAD-2*, *SMAD-4*, *α-SMA*, and *MMP-1* in ethanol-administered SD rats. * *p* < 0.05 vs. N.A.; ^#^ *p* < 0.05 vs. EtOH.

To investigate OEFL’s effect related to the ethanolic liver damage recovery, the change in the expressions levels of transforming growth factor-β (*TGF-β*), *SMAD-2*, *SMAD-4*, *α-SMA,* and *MMP-1* were measured in the liver tissues obtained from the experimental groups of SD rats (Figure 9). The expressions of *TGF-β*, *SMAD-2*, *SMAD-4*, and *α-SMA* increased by 1.2, 1.8, 1.1, and 1.1-fold, respectively, in the ethanol-administered group than in the normal group. Compared with the ethanol-administered group, the expressions of these genes were significantly reduced in the 200 and 600 mg/kg OEFL-administered groups. The expressions of *TGF-β*, *SMAD-2*, *SMAD-4*, and *α-SMA* decreased by 1.7, 1.8, 2.4, and 1.2-fold, respectively, in the test group administered with 3.6 mg/kg *p*-coumaric acid compared with the ethanol-administered group, confirming the possibility of collagen synthesis inhibition in the liver [36]. Therefore, this experiment demonstrated that OEFL and its main bioactive substance, *p*-coumaric acid, inhibited the collagen synthesis by inhibiting the *TGF-β/SMAD*-mediated signaling pathway and simultaneously decomposed the excessively synthesized collagen by activating *MMP-1*; expecting the inhibitory effect of ethanolic hepatocirrhosis [37].

## 3. Materials and Methods

### 3.1. Materials and Reagents

*O. javanica* was purchased from Nonghyup mart (Gochang, Jeonbuk, Korea) in June 2021 and dried at 60 °C using the forced convection dry oven (FC 49, Lab House, Seoul, Korea) for 24 h. Dried *O. javanica* were pulverized using a food grinder (Hanil HMF-3800, Seoul, Korea) and then passed through a 0.42 mm sieve. Powdered *O. javanica* was stored in polyethylene bag at 4 °C for later use. HPLC grade acetic acid and acetonitrile used for substance analysis of *O. javanica* were purchased from Thermo Fisher Sci., Inc. (Waltham, MA, USA). Deionized water (≥ 18 Ω) was produced in the laboratory using a Milli-Q purification system (Millipore, Burlington, VT, USA). 

### 3.2. Preparation of Extract

The bioactive substances were extracted from *O. javanica* using a UAE [38]. Powdered *O. javanica* (1 g) was placed into a pressure vessel with 10 mL of the 50% ethanol and mixed using a vortex mixer (VM-10, Daihan Scientific Co., Ltd., Wonju, Korea) for 1 min. Then, UAE was conducted using an ultrasound device (SD-250H, Mujigae Co., Seoul, Korea) at 60 °C for 30 min. The OE was centrifuged at 10,000 rpm for 10 min (236R, Labogene, Seoul, Korea). After centrifugation, the supernatant was separated from the mixture and filtered through a 0.45 μm syringe filter prior to analysis.

### 3.3. Microorganism Isolation and Identification

For microorganism screening, 1.5% agar was added to MRS to prepare an agar plate. Subsequently, 1.0 mL of kimchi broth diluted 100-fold was applied to agar and cultured at 37.0 °C for 24 h. A cultured single colony was harvested from the agar plat, inoculated into 10 mL of MRS broth, and incubated for 48 h. Following this, activities of 18 different enzyme activities were tested using the analytical profile index (API) kit (BioMerieux Co., Lyon, France), and strains with the highest *β*-glucosidase activity were selected. 

After DNA extraction of the selected strain, the 16S rRNA gene domain was amplified using PCR (MJ Research, PTC 225, MA, USA) to confirm chromosomes for strain identification using 27F (5′-AGA GTT TGA TCM TGG CTC AG-3′) and 1492R (5′-TAC GGY TAC CTT GTT ACG ACT T-3′) primers. The annealing temperature was set at 59.1 °C, and the amplified PCR product was purified using a QIAquick PCR purification kit (Qiagen, NRW, Germany). The analyzed nucleotide sequence was identified as *Lactiplantibacillus plantarum,* which showed the highest similarity compared with the nucleotide sequences registered in a Blast (US National Library of Medicine, Bethesda, USA).

### 3.4. Bioconversion Using L. plantarum

In the fermentation of *L. plantarum* for the production of enzymes used in bioconversion, the initial pH of MRS medium was adjusted to 7.0 using 1 M HCl and 1 M NaOH. The prepared medium was sterilized at 121 °C for 15 min in an autoclave (SAC05060P, Daihan Sci., Gangwon-do, Korea) and was used as a culture medium after cooling. After inoculation of 1.0% of *L. plantarum* into MRS medium, it was cultivated at 37 °C and 200 rpm for 24 h in a shaking incubator. For bioconversion, the UAE product and the culture broth were mixed in a volume ratio of 1:2 and further cultured at 37 °C and 200 rpm for 48 h. In other to produce crude enzymes, cell wall destruction was performed using a homogenizer (JSAT-65 JSR, Hanbaek Sci. Co., Bucheon, Korea) to obtain intracellular enzymes present in the cell. After the pH of the crude enzyme broth was adjusted to pH 5.0 ± 0.1 using 1 M HCl and 1 M NaOH, bioconversion by crude enzyme was induced at 45 °C and 200 rpm for 48 h. Then, the bioconverted broth was centrifuged at 4 °C and 5000 rpm for 10 min, and the supernatant was separated for the experiments.

### 3.5. HPLC Analysis

The HPLC used in the analysis was an Agilent Technologies 1260 equipped with a dual gradient pump, autosampler, column compartment, and diode-array detector (DAD). As an HPLC column, a Zorbax SB C18 column specialized for polyphenol analysis was used (4.6 × 150 mm, Agilent Technologies, Santa Clara, CA, USA) and the column temperature was maintained at 30 °C. The mobile phase was composed of A (1.0% acetic acid in water) and B (99.9% acetonitrile) with a gradient elution as follows: 0–5 min, 0.0–15.0% B; 5–50 min, 15–50% B; 50–60 min, 50–100% B; 60–64 min, 100–0.0% B. The flow rate and the injection volume were set at 0.5 mL/min and 10 μL, respectively. The individual polyphenol in the OE and the OEFL was identified by comparing its retention time as well as DAD spectra (190 to 640 nm) with those of polyphenol standards.

### 3.6. Experimental Animal Models

Experimental SD rats were 6-week-old male rats purchased from Orient Co., Ltd. (Seongnam, Korea), acclimatized constant feeding, temperature, humidity, and light conditions for 7 days. All experimental protocols were approved by the Institutional Animal Care and Use Committee of Jeonbuk National University (Jeonju, Korea; JBUH-IACUC-2021-36-1). During the acclimatization period, the weights of SD rats were measured at 10 a.m. every morning. Then, eight SD rats each were randomly divided into five different groups according to treatments, including normal group, ethanol administered group, ethanol +200 mg OEFL administered group, ethanol +600 mg OEFL administered group, and ethanol + *p*-coumaric acid administered group. As a diet, powdered feed for laboratory SD rats was supplied (Orient Bio Inc., Seongnam, Korea), and the SD rats had free access to water and feed. 

Prior to the experiment, to remove interference with the absorption of *ethanol* due to feed intake, the SD rats were fasted for 16 h, with unlimited access to water. The dosage (mg/kg) of the experimental substances was calculated based on the weight measured prior to the start of the test. The experimental substances including OEFL and *p*-coumaric acid were orally administered once 30 min before *ethanol* administration. The normal group and the *ethanol*-administered group were administered with distilled water instead of the experimental substance. 3 g/kg (30%) of ethanol was orally administered to all groups except the control group. The experimental substance was given intraoral by feeding directly into the stomach of the rats. During the experimental period, all SD rats were observed once daily (before and after administration) for checking health, general symptoms.

### 3.7. Blood and Liver Tissue Sampling

To analyze the biochemical changes in the blood caused by the administration of ethanol and experimental substance, venous blood sampling of the experimental SD rats was conducted at 0.5, 1, 2, and 4 h after ethanol administration. After completion of the experiment, rats fasted for 14 h. The collected blood was centrifuged at 3000 rpm for 10 min. The serum was separated and stored at −80 °C until analysis. SD rats were euthanized after drawing blood, and their livers were extracted. The liver was washed with saline solution and removed using filter paper. Thereafter, the liver was weighed and stored at −80 °C until analysis. 

### 3.8. Blood Ethanol Concentration Analysis

In each group of experimental SD rats, an ethanol assay kit (Megazyme Co., Wicklow, Ireland) was used to measure the change in blood ethanol concentration over time (0, 0.5, 1, 2, and 4 h). According to the manufacturer’s protocol, 10 μL of blood and 200 μL of reagent 1 (assay buffer, NAD, and ALDH mix) were mixed, and absorbance (A_1_) was measured at 340 nm. After 3 min, 50 μL of reagent 2 (ADH) was added to perform an additional reaction for 10 min, after which the absorbance (A_2_) of the solution was measured. After testing the standard solution in the same way as the blood, a calibration curve for the ethanol standard solution was generated based on the change in the absorbance (A_2_ − A_1_). Following this, the amount of ethanol present in the blood of SD rats of each group was calculated. 

### 3.9. Blood Aldehyde Concentration Analysis

The change in the concentration of blood acetaldehyde over time (0, 0.5, 1, 2, and 4 h) in the SD rats of each group was measured using an acetaldehyde quantification assay kit (Megazyme Co., WK, Ireland) according to the manufacturer’s protocol. 50 μL of blood was mixed with 1.0 mL of distilled water, 0.1 mL of azide (0.02% *w*/*v*), and 0.1 mL of nicotinamide adenine dinucleotide (NAD), and the absorbance (A_1_) was measured at 340 nm after reaction at room temperature for 2 min. Then, 25 μL of aldehyde dehydrogenase was added to the reaction solution, followed by reaction at room temperature for 5 min. The increased absorbance (A_2_) caused by NADH formation was measured at 340 nm. Blood ethanol and acetaldehyde concentrations were calculated based on the changing absorbance at 340 nm (A_2_ − A_1_). 

### 3.10. Blood GPT and GOT Concentrations Analysis 

To analysis blood GPT concentration, a diagnostic kit was purchased from Asan Pharmacy Co., Ltd. (Seoul, Korea) and testing was conducted with the manufacturer’s protocol. 1.0 mL of alanine, a substrate, was added to a tube and left at 37 °C for 5 min. Then, it was mixed with 0.2 mL of blood and reacted at 37 °C for 30 min. In addition, 1.0 mL of dinitrophenylhydrazine was added as a reagent to this solution. After 20 min, 10 mL of NaOH solution was added. Then, the reaction was performed at room temperature for 10 min, and absorbance was measured at 505 nm. The GPT concentration was calculated based on the calibration curve using lithium.

GOT analysis was performed by colorimetric method using a GOT diagnostic kit manufactured by Asan Pharmacy Co., Ltd. After adding 1.0 mL of L-aspartic acid as a substrate to 0.2 mL of blood, the reaction was performed at 37 °C for 60 min. After stopping the reaction, 10 mL of NaOH was added and then absorbance was measured at 505 nm. The GOT concentration is obtained from the standard curve using the pyruvate.

### 3.11. Blood Albumin Concentration Analysis

The albumin concentration was analyzed using a diagnostic kit (AM127, Asan Pharmaceutical Co., Ltd., Seoul, Korea) according to Bromocresol green method [39]. Approximately 5 mL of the reagent (Bromocresol green) was added to the blood collected at each sampling point from each group and the blood was reacted for 10 min. Then, absorbance was measured at 630 nm. The albumin concentration was calculated by creating a calibration curve using bovine serum albumin as a standard.

### 3.12. Blood ALP Analysis

Blood ALP concentration was analyzed using the industrial diagnostic kit (AM105S, Asan Pharmaceutical Co., Ltd.). 2 mL of buffer solution was reacted with 0.05 mL of blood and was further reacted for another 15 min. After that, 2 mL of a coloring reagent (sodium metaperiodate) was added and the reaction was performed for 10 min. The absorbance was then measured at 500 nm. According to the calibration curve of the standard solution, the blood ALP concentration was calculated. 

### 3.13. Reverse Transcription Polymerase Chain Reaction (RT-PCR)

RT-PCR was performed to determine the mRNA levels of *ADH, ALDH*, *TGF-β*, *SMAD-2*, *SMAD-4*, *α-SMA,* and *MMP-1* in the liver of rats. After homogenizing the liver, the total RNA was extracted using AccuPrep^®^ universal RNA extraction kit (Bioneer Co., Daejeon, Korea) and quantified using NanoDrop™ 2000c spectrophotometer (Thermo Fisher Sci., Inc. Waltham, MA, USA). Reverse transcription was performed for cDNA synthesis using the amfiRivert cDNA synthesis platinum master mix (GenDEPOT Co., Katy, TX, USA). The cDNA was amplified with each primer *ADH*, *ALDH*, *TGF-β*, *SMAD-2*, *SMAD-4*, *α-SMA*, *MMP-1*, and *β*-*actin* (Table 1). Each PCR product was electrophoresed on 1.0% agarose gel and visualized by using Gel Doc TM XR+system and quantified the expression of bands using Quantity One software (Bio-Rad Co., Hercules, CA, USA). 

## 4. Conclusions

In this study, a bioconversion of *O. javanica* extract using *L. plantarum* derived from kimchi was applied to improve the effect on a hangover relief and the recovery of ethanol-induced liver damage. A bioconversion product was produced that reduced blood ethanol and acetaldehyde concentrations while simultaneously improving ethanol-induced hepatocirrhosis.

HPLC analysis of OE and OEFL confirmed that chlorogenic acid, the main bioactive substance of OE, was converted to quinic acid. In addition, a new peak that did not exist in OE was detected in OEFL, and *p*-coumaric acid, detected as a main bioactive substance in OEFL, was 6.01 mg/g, an increase of 9.5-fold then OE, confirming that bioconversion using *L. plantarum* was effective. The SD rat models confirmed that in the 600 mg/kg OEFL and 3.6 mg/kg *p*-coumaric acid administered experimental groups, the blood ethanol, acetaldehyde, GPT, and ALP concentrations decreased compared with the ethanol-administered group, whereas albumin concentrations increased. In addition, to investigate the mechanism of effect related to hangover relief and improvement of ethanol-induced liver damage of OEFL and *p*-coumaric acid, the expressions levels of *ADH*, *ALDH*, *TGF-β*, *SMAD-2*, *SMAD-4*, *α-SMA*, and *MMP-1* in the liver were measured. *ADH*, *ALDH*, and *MMP-1* showed increased expression, and *TGF-β*, *SMAD-2*, *SMAD-4*, and *α-SMA* showed decreased expression, confirming that OEFL was effective in improving ethanol-induced liver damage through promoting ethanol oxidation. 

Therefore, the study proposes the possibility that OEFL and its bioactive substances, such as *p*-coumaric acid, can be used as food or medicine for the prevention and treatment of hangover and ethanol-induced liver damage.

## Figures and Tables

**Table 1:** List of primers used to determine gene expressions of *ADH, ALDH*, *TGF-β*, *SMAD-2*, *SMAD-4*, *α-SMA,* and *MMP-1* using RT-PCR. The sequence of designed primers for each gene is shown as forward and reverse.

Primer	Forward (5′–3′)	Reverse (5′–3′)
^1^ *ADH*	CGAGGGAGCTGGCATTGTT	CGGGTGCTTGCAGATCCT
^2^ *ALDH*	GCTGTGAAGGCCGCAAGA	GCCAGCAGCAGACGATCTC
^3^ *TGF-β*	CCCTGCCCCTACATTTGGA	GCACGCAGCACGGTGAT
*SMAD-2*	TGTGCAGAGCCCCAACTGT	CTGAGCCAGAAGAGCAGCAA
*SMAD-4*	CGACGCTGTGGGAAATGC	CTCCTCGCTGCGGTTCTG
^4^ *α-SMA*	TCCAGGGCTCCAACGAGAT	CCCCAAGTTCCGGTGTGA
^5^ *MMP-1*	CTGAAAAGCTGAGGCAAATGC	TGGTCCAACGAGGATTGTTGT
*β-actin*	CCCTGGCTCCTAGCACCAT	GAGCCACCAATCCACACAGA

^1^*ADH*: ethanol dehydrogenase; ^2^*ALDH*: aldehyde dehydrogenase; ^3^*TGF-β*: transforming growth factor-beta; ^4^*α-SMA*: α-smooth muscle actin; ^5^*MMP-1*: matrix metalloproteinase-1.

## Data Availability

No new data were created or analyzed in this study. Data sharing does not apply to this article.
